# Transcriptional dysregulation of autophagy in the muscle of a mouse model of Duchenne muscular dystrophy

**DOI:** 10.1038/s41598-024-51746-9

**Published:** 2024-01-16

**Authors:** Ryuta Nakashima, Ryusuke Hosoda, Yuki Tatekoshi, Naotoshi Iwahara, Yukika Saga, Atsushi Kuno

**Affiliations:** 1 Department of Pharmacology, Sapporo Medical University School of Medicine, South-1, West-17, Chuo-ku, Sapporo, 060-8556 Japan; 2 Department of Neurology, Sapporo Medical University School of Medicine, Sapporo, Japan

**Keywords:** Autophagy, Mechanisms of disease

## Abstract

It has been reported that autophagic activity is disturbed in the skeletal muscles of dystrophin-deficient *mdx* mice and patients with Duchenne muscular dystrophy (DMD). Transcriptional regulations of autophagy by FoxO transcription factors (FoxOs) and transcription factor EB (TFEB) play critical roles in adaptation to cellular stress conditions. Here, we investigated whether autophagic activity is dysregulated at the transcription level in dystrophin-deficient muscles. Expression levels of autophagy-related genes were globally decreased in tibialis anterior and soleus muscles of *mdx* mice compared with those of wild-type mice. DNA microarray data from the NCBI database also showed that genes related to autophagy were globally downregulated in muscles from patients with DMD. These downregulated genes are known as targets of FoxOs and TFEB. Immunostaining showed that nuclear localization of FoxO1 and FoxO3a was decreased in *mdx* mice. Western blot analyses demonstrated increases in phosphorylation levels of FoxO1 and FoxO3a in *mdx* mice. Nuclear localization of TFEB was also reduced in *mdx* mice, which was associated with elevated phosphorylation levels of TFEB. Collectively, the results suggest that autophagy is disturbed in dystrophin-deficient muscles via transcriptional downregulation due to phosphorylation-mediated suppression of FoxOs and TFEB.

## Introduction

Duchenne muscular dystrophy (DMD), which is caused by mutation of *DMD* gene encoding dystrophin, is an X-linked disease with poor clinical outcomes^1^. DMD is characterized by degeneration, necrosis, and skeletal muscle regeneration, resulting in progressive muscle weakness and atrophy. The general treatment of DMD is the use of a corticosteroid. Recently, exon skipping has been used as a new therapy for patients with DMD. However, exon skipping has limitations such as limited delivery of the drug to targeted tissues after administration and the inability to address all pathogenic mutations in the gene^2^. Therefore, there is a need to develop novel therapies based on the understanding of molecular mechanisms of the disease.

Autophagy is an intracellular system to degrade damaged proteins and organelles for recycling and is essential for cell survival^3^. In skeletal muscles, autophagy plays a critical role in the maintenance of muscle mass. Muscle-specific deletion of Atg7, an essential autophagy effector gene, causes muscle atrophy^4^. Overexpression of Atg5, a gene involved in phagophore elongation, improves the age-related decline in muscle strength of mice^5^. The process of autophagy is controlled by nutrition-sensitive signals. It has been reported that mammalian target of rapamycin complex 1 (mTORC1), activated in a rich-nutrient condition^6^, inhibits autophagosome formation by phosphorylating ULK1^7,8^, Atg13^8^, and Atg14L^9^. mTORC1 also negatively regulates a step of autophagosome-lysosome fusion via phosphorylation of UVRAG^10^. AMPK has been shown to be a positive regulator of autophagosome formation through phosphorylation of ULK1^7^. Autophagy is also regulated at the transcriptional level. FoxO transcription factors (FoxOs) have been reported to positively regulate the expression of autophagy-related genes^11–14^. Phosphorylation of FoxOs by Akt promotes their nuclear export, thereby inhibiting their transcriptional activity^15,16^. Transcription factor EB (TFEB) also promotes the expression of autophagy-related and lysosomal genes^17–19^. Transcriptional activity of TFEB is also controlled by its phosphorylation status. Phosphorylation of TFEB by mTORC1^20,21^ promotes its nuclear export and suppresses its transcriptional activity, whereas TFEB is dephosphorylated via calcineurin activated by MCOLN1-mediated calcium signaling^22^.

It has been reported that autophagic activity in the skeletal muscle is reduced in DMD patients and in *mdx* mice, a model of DMD^23–25^. De Palma et al.^23^ and we^24^ reported that the Akt-mTORC1 signal is enhanced in skeletal muscles from DMD patients and *mdx* mice. Restoration of autophagy by treatment of *mdx* mice with rapamycin, an inhibitor of mTORC1, led to an increase in muscle strength and an improvement of cardiac function^26^. These findings suggest that an enhanced Akt-mTORC1 signal is responsible for impaired autophagy and muscular and cardiac damage in DMD. In addition, it has been reported that the expression levels of autophagy-related genes are decreased in quadriceps, diaphragm, and tibialis anterior muscles in *mdx* mice^23,24^. We reported that treatment of *mdx* mice with resveratrol, an activator of the NAD^+^-dependent protein deacetylase SIRT1^27^, restores the expression of autophagy-related genes and ameliorates skeletal muscle injury^24,28^. Activation of TFEB via MCOLN1 has been reported to attenuate muscle damage in *mdx* mice^29^. These reports suggest that repression of autophagy-related genes contributes to suppressed autophagic activity and muscle injury in *mdx* mice. However, it remains unknown how autophagy-related genes are downregulated in DMD. Although it has been reported that nucleus-localized TEFB is reduced in the diaphragm of *mdx* mice^30^, it is unknown whether its transcriptional activity is downregulated via mTORC1-mediated phosphorylation in *mdx* mice.

In this study, we investigated whether transcription of autophagy-related genes is dysregulated in the skeletal muscle of *mdx* mice. We focused on FoxOs and TFEB, because Akt-mTORC1 signaling, which leads to suppression of those transcription factors, is activated in dystrophic muscles.

## Results

### Autophagic activity is reduced in the skeletal muscle of *mdx* mice

**Figure 1:**
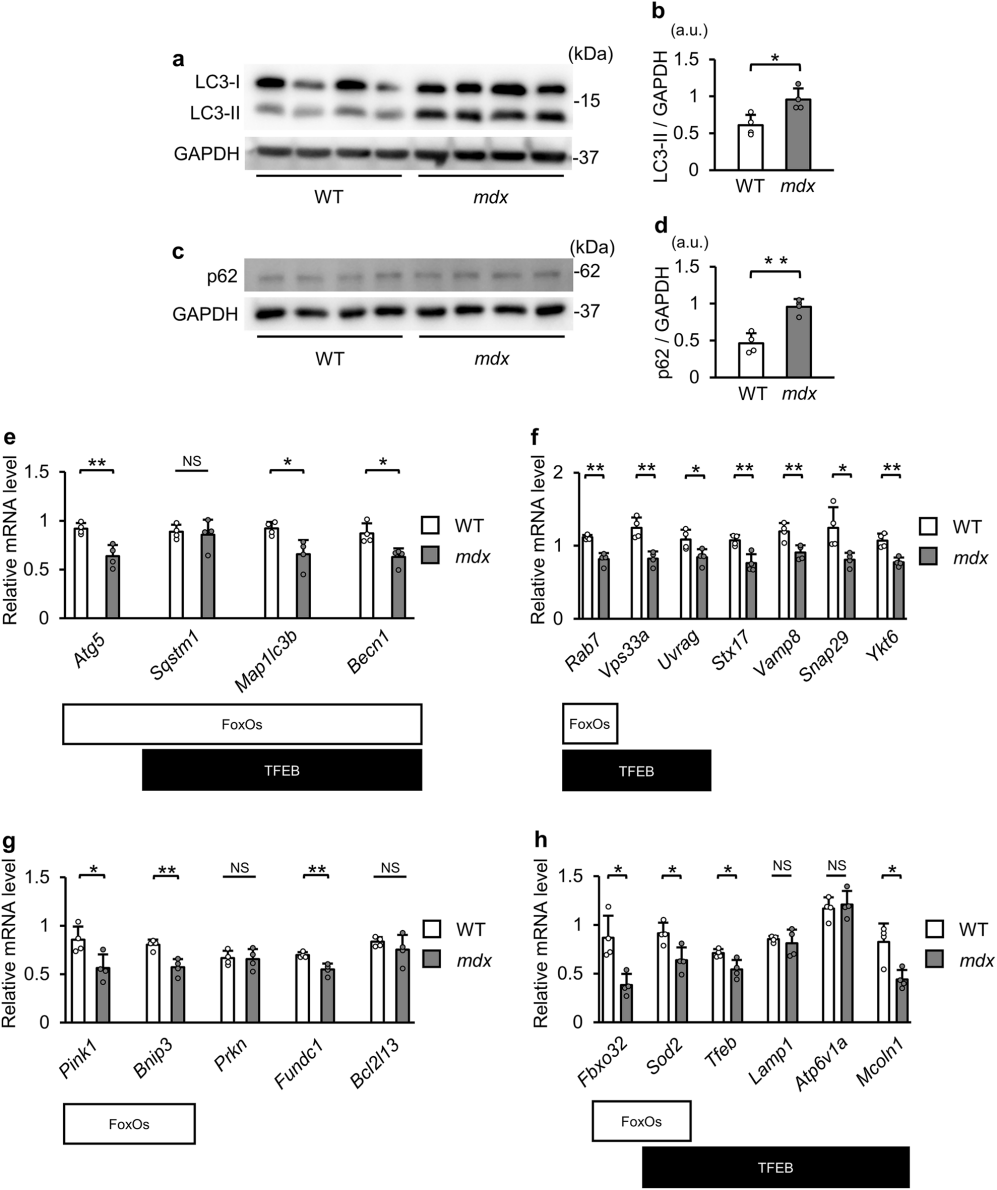
Autophagic activity is reduced and autophagy-related genes are downregulated in tibialis anterior muscles in *mdx* mice. (**a**) Representative Western blot images for LC3 and GAPDH in tibialis anterior muscles from WT mice and *mdx* mice at 22 weeks of age. kDa: kilodalton. (**b**) Quantitative data of LC3-II levels normalized to GAPDH (N = 4). (**c**) Representative Western blot images for p62 and GAPDH in tibialis anterior muscles from WT mice and *mdx* mice at 22 weeks of age. (**d**) Quantitative data of p62 levels normalized to GAPDH (N = 4). (**e–h**) Expression levels of genes related to autophagosome formation (**e**), the step of autophagosome-lysosome fusion (**f**), and mitophagy (**g**), and other target genes of FoxOs or TFEB (**h**) in tibialis anterior muscles of WT mice and *mdx* mice at 22 weeks of age (N = 4) examined by real-time quantitative PCR. Target genes of FoxOs and TFEB are indicated. All data are expressed as means ± standard deviation. All statistical tests were conducted by Welch’s two sample t-test. a.u. = arbitrary unit. *P < 0.05, **P < 0.01; NS, not significant.

We assessed the muscle strength of forearms in C57BL/10Sn mice as control wild-type (WT) and *mdx* mice at 12–13, 21–22, and 60–61 weeks of ages. As previously reported^31^, grip grasping power was lower in *mdx* mice than that in WT mice at those ages (Table 1). Because fast type fibers are firstly damaged in DMD patients^32^, we mainly analyzed tibialis anterior muscles at 22 weeks of age in this study. Western blotting showed that the protein level of LC3-II, an autophagosome marker, was significantly higher in the tibialis anterior muscle of *mdx* mice than in that of WT mice (Fig. 1a and b). The level of p62 protein, which is degraded by autophagy, was also higher in *mdx* mice (Fig. 1c and d). These data suggest that autophagosomes were accumulated via impaired autophagosome degradation in *mdx* mice.

**Table 1:** Grip strength and body weight in *mdx* mice.

(Weeks of age)	Grip strength (kg)		Body weight (g)	
	WT	*mdx*	WT	*mdx*
12–13	0.12 ± 0.01 (N = 10)	0.08 ± 0.02*** (N = 10)	29.3 ± 0.7 (N = 10)	27.8 ± 2.1 (N = 10)
21–22	0.14 ± 0.02 (N = 7)	0.08 ± 0.01*** (N = 7)	33.9 ± 1.5 (N = 7)	31.0 ± 1.0** (N = 7)
60–61	0.12 ± 0.02 (N = 7)	0.10 ± 0.01** (N = 6)	40.4 ± 2.1 (N = 7)	31.8 ± 3.6***(N = 6)

All data are expressed as means ± standard deviation. All statistical tests were conducted by Welch’s two sample t-test. **P < 0.01, and ***P < 0.001 versus corresponding age of WT.

### Expression levels of autophagy-related genes are decreased in *mdx* mice

Next, we investigated the expression levels of autophagy-related genes in the tibialis anterior muscle of mice at 22 weeks of age. We performed real-time quantitative PCR to measure transcriptional levels of genes. Expression levels of *Atg5*, *Sqstm1*, *Map1lc3b* and *Becn1*, which are involved in autophagosome formation^3^, were significantly lower in *mdx* mice than in WT mice (Fig. 1e). We also analyzed genes related to the fusion step of autophagosomes and lysosomes, including *Rab7*^33^, *Vps33a*^34^, *Uvrag*^10^, *Stx17*^35^, *Vamp8*^35,36^, *Snap29*^35,37^ and *Ykt6*^38^. Expression levels of these genes were decreased in *mdx* mice (Fig. 1f). Mitophagy is a type of autophagy that specifically targets damaged or dysfunctional mitochondria for their degradation. Mitophagy-related genes, *Pink1*, *Bnip3* and *Fundc1*, were downregulated in *mdx* mice (Fig. 1g). Among the genes mentioned above, FoxOs are known to regulate the expression of *Atg5*^14^, *Sqstm1*^12^, *Map1lc3b*^11,12^, *Becn1*^39^ (Fig. 1e), *Rab7*^13^ (Fig. 1f), *Pink1*^40^ and *Bnip3*^11,12^ (Fig. 1g). Additionally, the levels of *Sod2* and *Fbxo32*, target genes of FoxOs^41,42^ were reduced in *mdx* mice (Fig. 1h), suggesting reduced transcription activity of FoxOs. It has been reported that TFEB regulates the expression of *Sqstm1*^17,18^, *Map1lc3b*^18^, *Becn1*^17^ (Fig. 1e), *Rab7*^17^, *Vps33a*^17^, *Uvrag*^17,18^ (Fig. 1f), and *Sod2*^43^ (Fig. 1h). Although mRNA levels of *Tfeb*^44^ and *Mcoln1*^17,19^, TFEB target genes, were reduced in *mdx* mice, levels of the other targets of TFEB, *Lamp1*^17,19^ and *Atp6v1a*^17^, were unchanged (Fig. 1h). These data suggest that transcriptional regulation of autophagy-related genes was suppressed via reduced activity of FoxOs and TFEB in tibialis anterior muscles of *mdx* mice.

We also examined the expression levels of autophagy-related genes in the soleus muscle, which mainly contains a slow type of muscle fibers, in mice at 22 weeks of age. We found that many autophagy-related genes were downregulated in the soleus muscle of *mdx* mice as observed in the tibialis anterior muscle (Supplementary Fig. S1).

Furthermore, we also analyzed autophagy-related genes in tibialis anterior muscles of mice at a younger age of 12 weeks (Supplementary Fig. S2). The expression levels of *Map1lc3b*, *Becn1*, *Uvrag*, *Bnip3*, *Lamp1*, and *Fbxo32* were lower in *mdx* mice, suggesting suppressed activities of FoxOs and TFEB even at 12 weeks of age.

### Changes in expression levels of autophagy-related genes in skeletal muscles from patients with DMD

**Figure 2:**
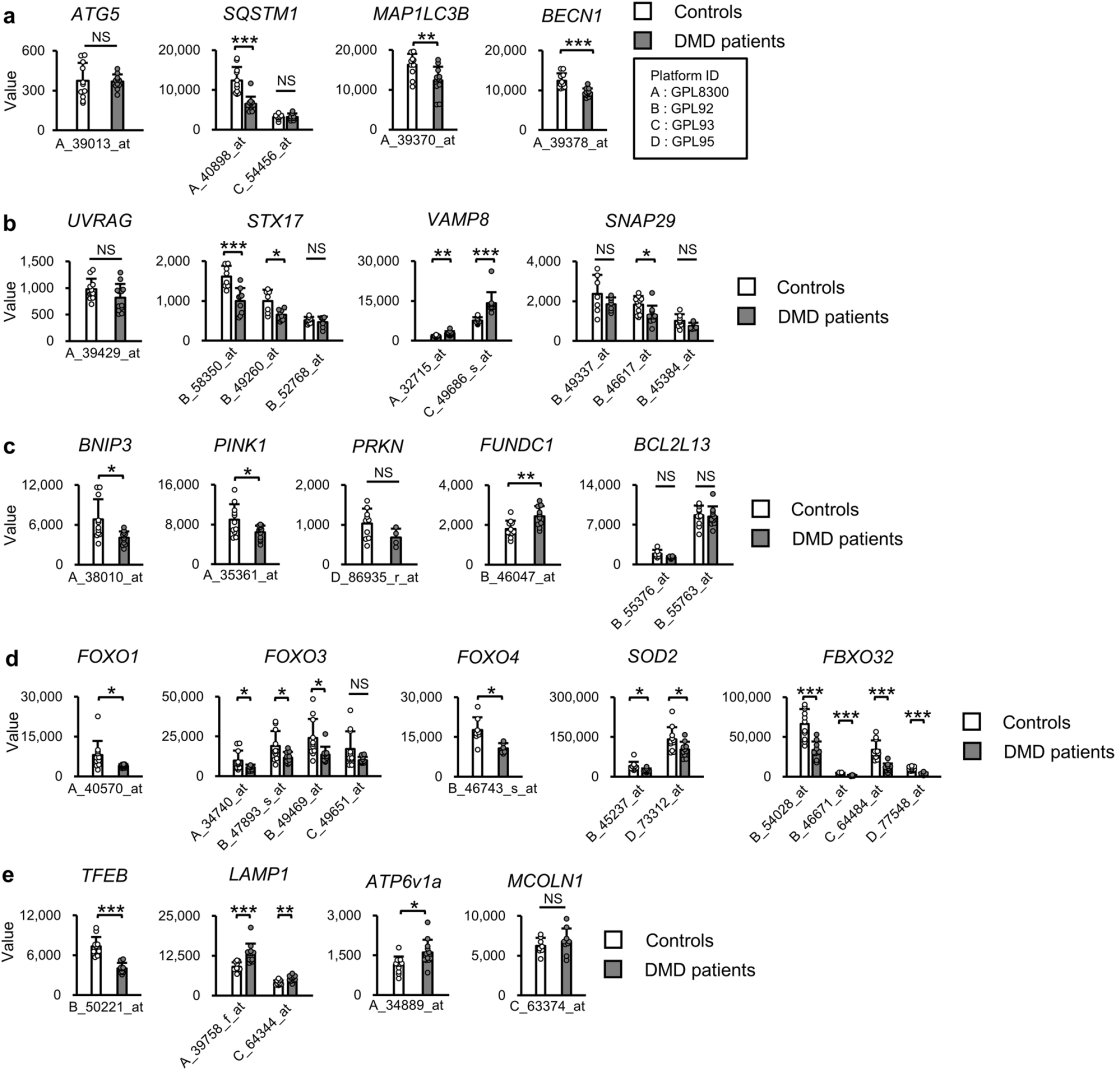
Expression levels of autophagy-related genes in muscles of patients with Duchenne muscular dystrophy. Expression levels of genes related to autophagosome formation (**a**), the step of autophagosome-lysosome fusion (**b**), and mitophagy (**c**), and FOXO family and genes regulated by FOXOs (*SOD2, FBXO32*) (**d**) and target gene of TFEB (*TFEB, LAMP1, ATP6V1a, and MCOLN1*) (**e**) in quadriceps muscles of healthy controls and patients with Duchenne muscular dystrophy (DMD). Microarray datasets were obtained from Gene Expression Omnibus (https://www.ncbi.nlm.nih.gov/geo/). All data are expressed as means ± standard deviation. All statistical tests were conducted by Welch’s two sample t-test. *P < 0.05, **P < 0.01, ***P < 0.001; NS, not significant.

We analyzed DNA microarray data of biopsy specimens from quadriceps muscles^45,46^ to compare the expression levels of autophagy-related genes in healthy controls and DMD patients. Expression levels of *SQSTM1* (GSE1004_GPL8300_40898_at), *MAP1LC3B* (GSE1004_GPL8300_39370_at) and *BECN1* (GSE1004_GPL8300_39378_at) were decreased in DMD patients (Fig. 2a). Expression levels of *STX17* (GSE1007_GPL92_58350_at, GSE1007_GPL92_49260_at) and *SNAP29* (GSE1007_GPL92_46617_at) were also reduced in DMD patients (Fig. 2b). In addition to genes listed above, *BNIP3* (GSE1004_GPL8300_38010_at) and *PINK1* (GSE1004_GPL8300_35361_at) were downregulated in DMD (Fig. 2c). Expression levels of *FOXO1* (GSE1004_GPL8300_40570_at), *FOXO3* (GSE1004_GPL8300_34740_at, GSE10007_GPL92_47893_at, GSE1007_GPL92_49469_at, and GSE1007_GPL93_49651_at), *and FOXO4* (GSE1007_GPL92_46743_s_at) were decreased in DMD patients (Fig. 2d). Target genes of FOXOs, *SOD2* (GSE1007_GPL92_45237_at, GSE1007_GPL95_73312_at) and *FBXO32* (GSE1007_GPL92_54028_at, GSE1007_GPL92_46671_at, GSE1007_GPL93_64484_at, GSE1007_GPL95_77548_at), were also downregulated (Fig. 2d). These findings indicate reductions in transcriptional activities of FOXOs in patients with DMD. Expression levels of TFEB (GSE1007_GPL92_50221_at) and its target genes (*SQSTM1, MAP1LC3B*, *or BECN1*) were decreased in DMD patients (Fig. 2a and e), although *LAMP1*, *ATP6V1a* and *MCOLN1*, were not reduced in DMD (Fig. 2e).

### FoxOs localized in the nucleus are reduced in *mdx* mice

**Figure 3:**
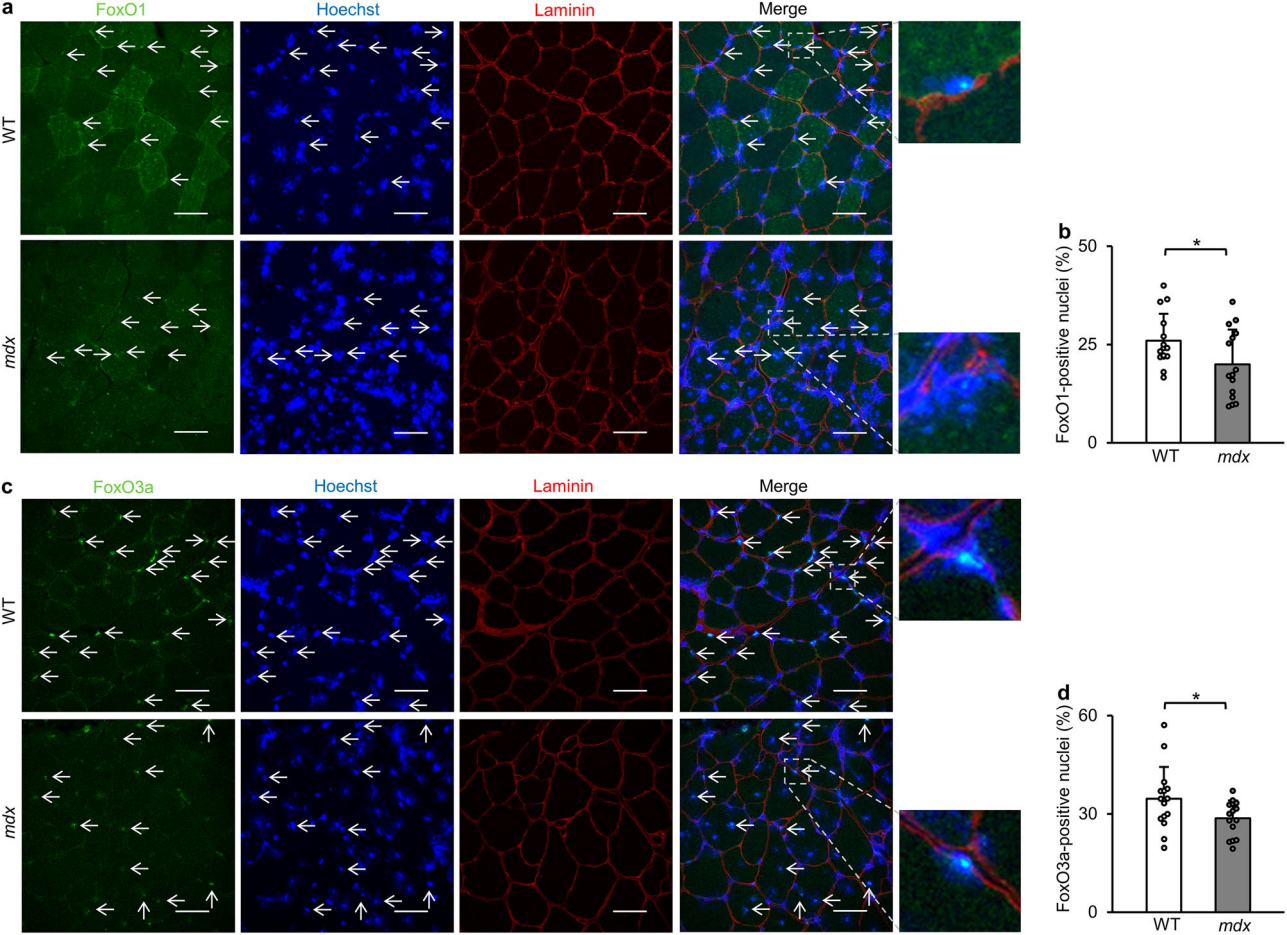
Nuclear localization of FoxO1 and FoxO3a is decreased in tibialis anterior muscles of *mdx* mice. (**a**) Representative images of immunofluorescence for FoxO1 (green) and laminin (red), nuclear staining with Hoechst33342 (blue) and merged images in tibialis anterior muscles of WT mice and *mdx* mice at 22 weeks of age. The arrow indicates FoxO1 co-localized with the nucleus (Hoechst33342). Scale bar: 50 μm. (**b**) Percentage of FoxO1-positive nuclei. We analyzed 15 randomly selected images from 3 mice (N = 15 in each group). (**c**) Representative images of immunofluorescence for FoxO3a (green) and laminin (red), Hoechst33342 staining (blue) and merged images in tibialis anterior muscle sections of WT mice and *mdx* mice. The arrow indicates FoxO3a localized in the nucleus. Scale bar: 50 μm. (**d**) Percentage of FoxO3a-positive nuclei. We analyzed 15 randomly selected images from 3 mice in each group (N = 15). All data are expressed as means ± standard deviation. All statistical tests were conducted by Welch’s two sample t-test. *P < 0.05.

Nuclear localization is essential for transcriptional activities of FoxOs. We therefore examined the localization of FoxO1 and FoxO3a in tibialis anterior muscles (Fig. 3a and c). Immunofluorescence showed that the percentage of FoxO1-positive nuclei was decreased in *mdx* mice compared with that in WT mice (Fig. 3a and b). FoxO3a localized in the nucleus was also reduced in *mdx* mice (Fig. 3c and d). These data collectively suggest that transcriptional activities of FoxO1 and FoxO3a were decreased in *mdx* mice.

### Phosphorylation levels of FoxOs are increased in *mdx* mice

**Figure 4:**
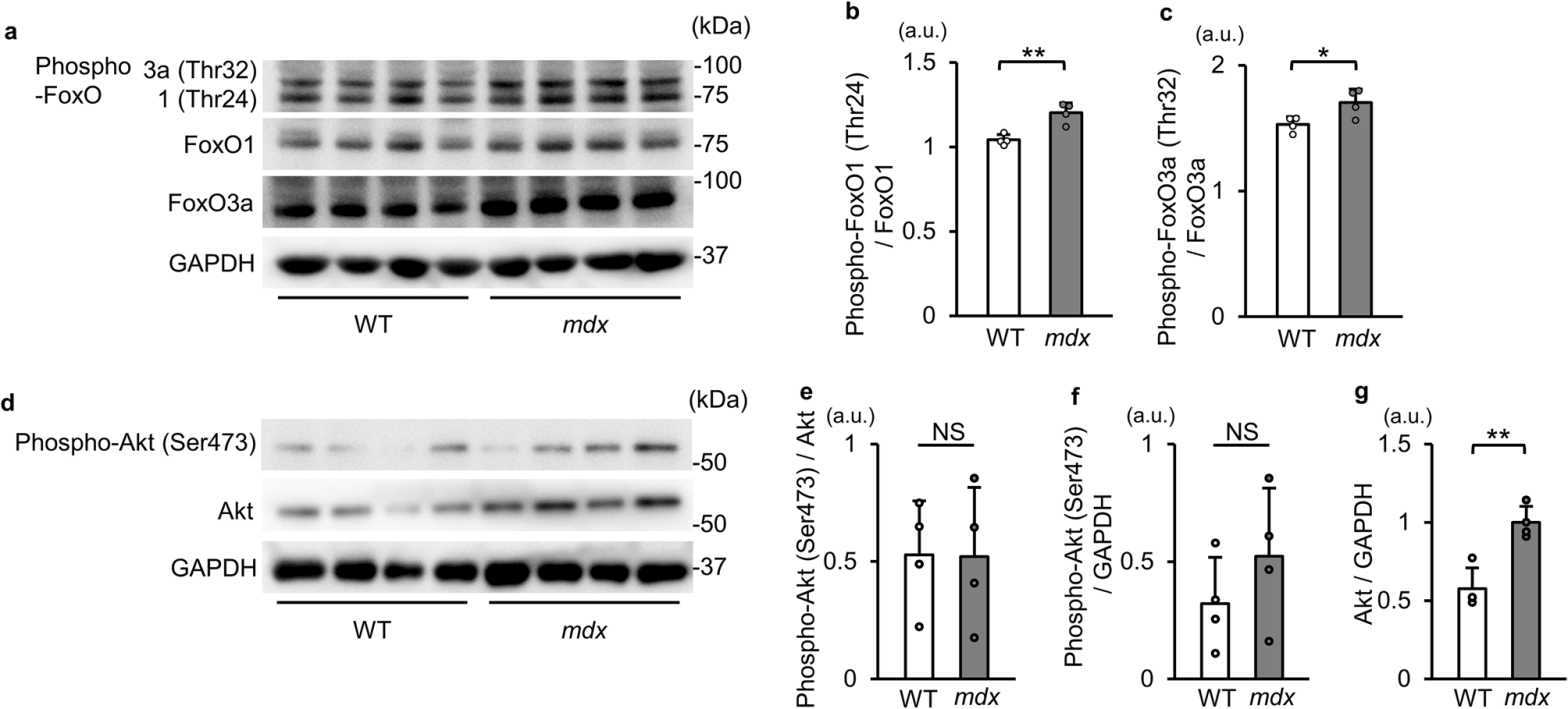
Phosphorylation levels of FoxO1 and FoxO3a are increased in tibialis anterior muscles of *mdx* mice. (**a**) Western blot images for phospho-FoxO1 (Thr24), phospho-FoxO3a (Thr32), FoxO1, FoxO3a and GAPDH in tibialis anterior muscles from WT mice and *mdx* mice at 22 weeks of age. kDa: kilodalton. (**b**) Quantitative data of phosphorylation levels of FoxO1 normalized to FoxO1 (N = 4). (**c**) Quantitative data of phosphorylation levels of FoxO3a normalized to FoxO3a (N = 4). (**d**) Western blot images for phospho-Akt (Ser473) and total Akt in WT and *mdx* mice. (**e–g**) Quantitative data of phosphorylation and total levels of Akt normalized to GAPDH (N = 4). All data are expressed as means ± standard deviation. All statistical tests were conducted by Welch’s two sample t-test. a.u. = arbitrary unit. *P < 0.05, **P < 0.01; NS, not significant.

It has been reported that FoxOs are phosphorylated by Akt, and this phosphorylation leads to nuclear export of FoxOs and results in inhibition of their transcriptional activities^15,16^. Additionally, Akt has been reported to be activated in DMD patients and *mdx* mice^23^. We therefore investigated phosphorylation levels of FoxO1 and FoxO3a. Western blotting showed that phosphorylation levels of FoxO1 at Thr24 and FoxO3a at Thr32, phosphorylation sites by Akt^15,16^ were increased in *mdx* mice (Fig. 4a–c). Levels of both phospho-Ser473-Akt and total Akt were increased in *mdx* mice, although the increase in the phospho-Akt level did not reach statistical significance (Fig. 4d–g). These results suggest that nuclear localization of FoxO1 and FoxO3a was decreased via their increased phosphorylation in *mdx* mice.

To further investigate the involvement of phosphorylation of FoxOs, we analyzed *mdx* mice at 71 weeks of age. Phosphorylation levels of FoxO1 and FoxO3a were also increased in *mdx* mice at this age (Supplementary Fig. S3c–e). These changes were associated with the increase in LC3-II levels in *mdx* mice (Supplementary Fig. S3a and b). Collectively, these data strongly indicate critical roles of FoxO phosphorylation on the regulation of autophagy.

### Nuclear localization of TFEB is attenuated in *mdx* mice

**Figure 5:**
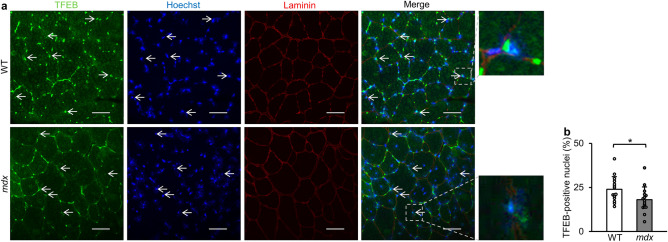
TFEB localized in the nucleus is reduced in tibialis anterior muscles in *mdx* mice. (**a**) Representative images of immunofluorescence staining for TFEB (green) and laminin (red), nuclear staining with Hoechst33342 (blue) and merged images in tibialis anterior muscles of WT mice and *mdx* mice at 22 weeks of age. The arrow indicates TFEB located in the nucleus. Scale bar: 50 μm. (**b**) Percentage of TFEB-positive nuclei. We analyzed 15 randomly selected images from 3 mice in each group (N = 15). All data are expressed as means ± standard deviation. All statistical tests were conducted by Welch’s two sample t-test. *P < 0.05.

We next performed immunofluorescence for TFEB with tibialis anterior muscles to investigate its intracellular localization in *mdx* mice. As expected, the number of TFEB-positive nuclei was significantly reduced in the tibialis anterior muscle of *mdx* mice (Fig. 5a and b), suggesting the reduction in TFEB activity in *mdx* mice.

### Phosphorylation levels of TFEB are increased in *mdx* mice

**Figure 6:**
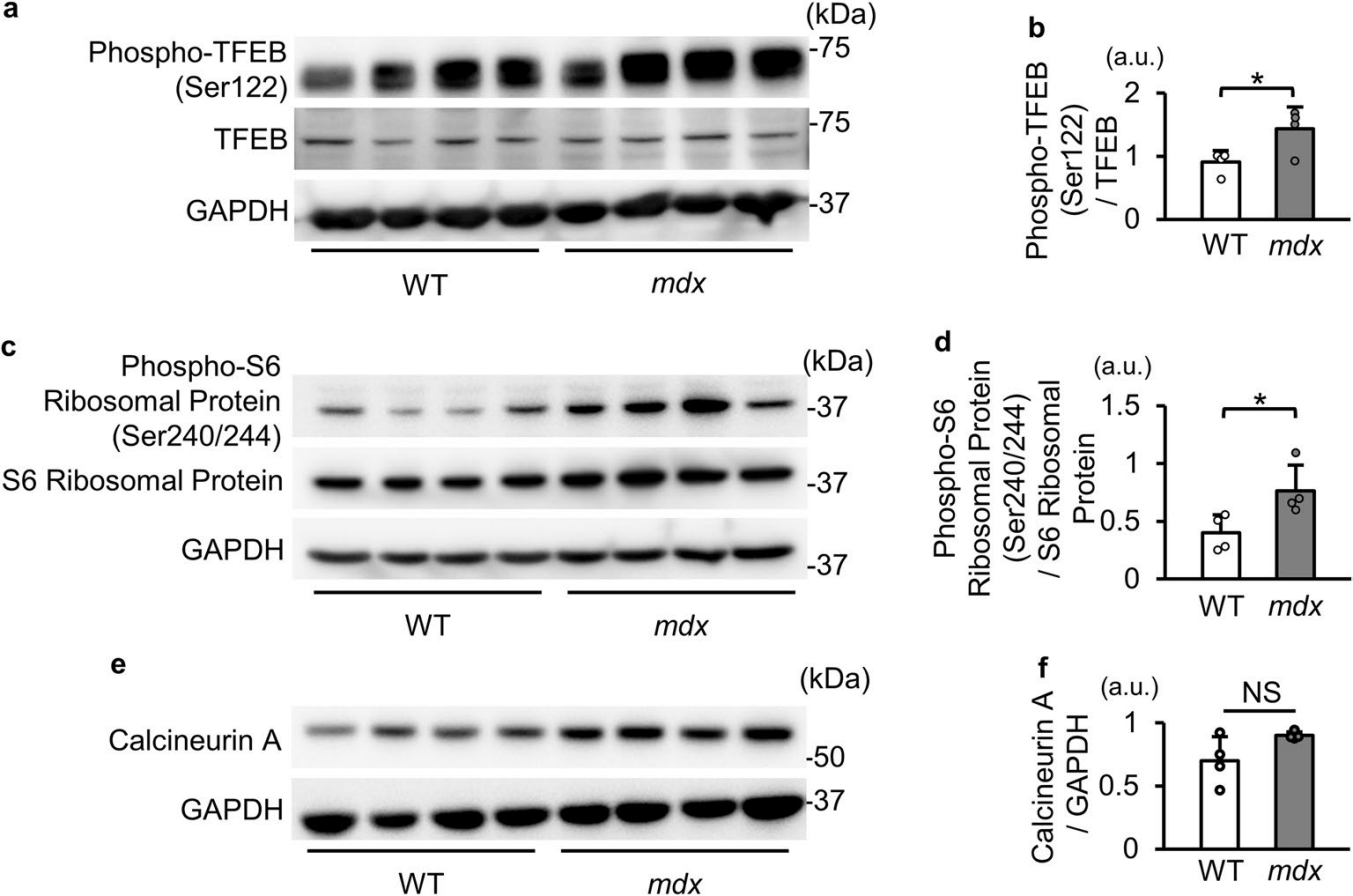
Phosphorylation levels of TFEB and S6 protein are increased in tibialis anterior muscles in *mdx* mice. (a) Representative Western blot images for phospho-TFEB (Ser122), TFEB and GAPDH in tibialis anterior muscles of WT mice and *mdx* mice at 22 weeks of age. kDa: kilodalton. (**b**) Quantitative data of phosphorylation levels of TFEB normalized to TFEB (N = 4). (**c**) Representative Western blot images for phospho-S6 ribosomal proteins (Ser240/244), S6 ribosomal protein and GAPDH in tibialis anterior muscles from WT mice and *mdx* mice at 22 weeks of age. (**d**) Quantitative data of phosphorylation levels of S6 ribosomal protein normalized to S6 ribosomal protein (N = 4). (**e**) Representative Western blot images for calcineurin A. (**f**) Quantitative data of calcineurin A normalized to GAPDH (N = 4). All data are expressed as means ± standard deviation. All statistical tests were conducted by Welch’s two sample t-test. a.u. = arbitrary unit. *P < 0.05; NS, not significant.

It has been reported that TFEB is phosphorylated by mTORC1, leading to its nuclear export and transcriptional inactivation^20,21^. Moreover, mTORC1 activity has been reported to be increased in *mdx* mice^23,24^. Based on the knowledge, we hypothesized that phosphorylation of TFEB is increased in *mdx* mice. The phosphorylation level of TFEB at Ser122, one of the phosphorylation targets of mTORC1^21^, was increased in *mdx* mice (Fig. 6a and b). As expected, phosphorylation level of S6 ribosomal protein, a downstream effector of mTORC1, at Ser240/244 was higher in *mdx* mice than in WT mice (Fig. 6c and d). The protein level of calcineurin A, which is the catalytic subunit of calcineurin known to dephosphorylate TFEB^22^, was not reduced in *mdx* mice (Fig. 6e and f). At 71 weeks of age, phospho-TFEB levels were also higher in *mdx* mice (Supplementary Fig. 3f and g). Protein levels of calcineurin A were comparable in WT and *mdx* mice (Supplementary Fig. S3f and h). These data suggest that TFEB activity was decreased in *mdx* mice via mTORC1-mediated phosphorylation and subsequent nuclear export.

### Resveratrol treatment restores autophagic activity and reduces phospho-TFEB level in tibialis anterior muscles of *mdx* mice

We previously reported that treatment of *mdx* mice with resveratrol, a known activator of the NAD^+^-dependent SIRT1, increases autophagic activity and expression levels of autophagy-related genes, including those that are targets of FoxOs and TFEB, in quadriceps muscles^24^. Additionally, in our earlier research, we observed an increase in the nuclear localization of FOXO3a as a result of resveratrol treatment in the hearts of *mdx* mice^47^. Furthermore, it has been documented that resveratrol treatment can lead to dephosphorylation of TFEB and its nuclear translocation in non-myocytes^48^. Therefore, we examined whether resveratrol treatment changes the phosphorylation status of FoxOs and TFEB in tibialis anterior muscles of *mdx* mice. Administration of resveratrol (0.4 g/kg food) to *mdx* mice was started at 9 weeks of age, and the muscle tissues were examined at 65 weeks of age as previously reported^24^. As observed in quadriceps muscles in our earlier study^24^, total LC3 (LC3-I + LC3-II) levels were reduced by resveratrol treatment, suggesting a restoration of autophagic activity (Supplementary Fig. S4a and b). Phosphorylation levels of FoxO1 and FoxO3 were not changed by resveratrol treatment (Supplementary Fig. S4c and d). On the other hand, phospho-TFEB levels were decreased by resveratrol treatment (Supplementary Fig. S4e and f). Resveratrol treatment did not change calcineurin A protein levels (Supplementary Fig. S4e and f).

## Discussion

The present study showed that genes related to the autophagic process were globally downregulated in muscles of *mdx* mice. Nuclear FoxO1 and FoxO3a levels were decreased in *mdx* mice, which were associated with increases in phosphorylation levels of FoxO1 and FoxO3a. In addition, phospho-TFEB level was increased and nuclear TFEB was decreased in *mdx* mice. These findings suggest downregulation of autophagy-related genes in muscles of *mdx* mice via phosphorylation-mediated reduction in activities of FoxOs and TFEB. Downregulation of these genes was also observed in muscles from patients with DMD. This is the first report showing transcriptional dysregulation of autophagy in dystrophin-deficient skeletal muscles.

The process of autophagy is separated into autophagosome formation, fusion of autophagosomes with lysosomes, and degradation of the contents of autophagosomes by lysosomal enzymes. In this study, we showed that levels of LC3-II, a marker of autophagosomes, were increased in the tibialis anterior muscle of *mdx* mice. Although transcript levels of p62 (*Sqstm1*) were similar in WT mice and *mdx* mice, p62 protein level was higher in *mdx* mice. These findings are consistent with results for the quadriceps muscle of *mdx* mice in our previous study^24^ and in the diaphragm in a study by Spaulding HR et al.^30^. Although genes related to autophagosome formation were also downregulated, suppression of autophagosome clearance was dominantly involved in the impaired autophagic activity in *mdx* mice because the autophagosome marker LC3-II were accumulated in Western blotting analysis. Since lysosomal genes (*Lamp1* and *Atp6v1a*) were not changed, the reduction in the expression levels of gene-related to autophagosome-lysosome fusion may have contributed to the suppression of autophagosome clearance in *mdx* mice. In addition, it is possible that activated mTORC1 also inhibits autophagosome-lysosome fusion by phosphorylating UVRAG^10^ and VAMP8^49^.

Here, we report for the first time that transcriptional activities of FoxOs are reduced probably via their enhanced phosphorylation in *mdx* mice. Although Akt, which is known to phosphorylate FoxOs^15,16^, has been reported to be activated in skeletal muscle of *mdx* mice^23^, the increase in phospho-Akt levels in *mdx* mice did not reach statistical significance in the present study. Therefore, we cannot exclude the possibility that another kinase is also responsible for phosphorylation of FoxOs. Serum- and glucocorticoid-inducible kinase (SGK) phosphorylates FoxOs at the same sites as Akt^50^. There has been no study in which SGK activity in skeletal muscle of *mdx* mice was investigated. However, since *mdx* mice that lack SGK1 showed favorable phenotypes of skeletal muscles^51^, SGK may be activated in *mdx* mice. The other possible regulation of intracellular localization of FoxOs is mediated by SIRT1. FoxOs are deacetylation targets of SIRT1^52^ and it has been reported that SIRT1 promotes nuclear localization of FoxOs^53,54^. Our previous study showed that administration of resveratrol, an activator of SIRT1, to *mdx* mice upregulated the expression levels of FoxO-regulated genes in skeletal muscle and the heart^24,47^. Furthermore, resveratrol treatment increased nuclear FoxO3a level in the heart of *mdx* mice^47^. Therefore, reduced SIRT1 activity may also be involved in nuclear exclusion of FoxOs in *mdx* mice.

Although nuclear TFEB was reported to be decreased in *mdx* mice^30^, the mechanism has not been determined. We found that the increase in phosphorylation level of TFEB at Ser122, a phosphorylation site by mTORC1, was associated with reduction in nucleus-localized TFEB in *mdx* mice. Since the phosphorylation level of S6, a downstream effector of mTORC1, was also elevated in *mdx* mice, it is possible that mTORC1-mediated TFEB phosphorylation led to its nuclear exclusion. Calcineurin dephosphorylates TFEB at Ser142 and Ser211 and increases transcriptional activity of TFEB^22^. However, calcineurin activity was reported to be unchanged in the tibialis anterior muscle and rather increased in the diaphragm of *mdx* mice at the age of 5 months^55^. Indeed, in the present study, the protein level of calcineurin A was not reduced in *mdx* mice. Therefore, it is unlikely that reduced dephosphorylation by calcineurin is responsible for increased phosphorylation of TFEB in *mdx* mice.

Since FoxOs and TFEB share many genes as targets, it is difficult to determine which transcription factors are dominantly affected in dystrophin-deficient muscles. Genes related to lysosomes such as *Lamp1* and *Atp6v1a*, which are main targets of TFEB, were not changed in the tibialis anterior muscles of *mdx* mice. Furthermore, we observed that expression levels of *LAMP1*, *ATP6V1a*, and *MCOLN1* in muscles of DMD patients were unchanged or rather upregulated despite a reduction in the expression of *TFEB*. These findings suggest that FoxOs, rather than TFEB, might play a dominant role in the downregulation of autophagy-related genes in the tibialis anterior muscle of *mdx* mice. In contrast, in the present study, expression levels *Tfeb* and *Lamp1* were downregulated in the soleus muscles. In our previous study, the expression level of *Tfeb* in the quadriceps of *mdx* mice was reduced by approximately 50% compared to that in control mice^24^. In addition, TFEB activity determined by using a protein-DNA array has been reported to be increased in the diaphragm muscle from 6-week-old *mdx* mice^56^. Therefore, these findings suggest that changes in TFEB expression and activity may vary depending on skeletal muscle types.

One limitation of this study is that there was no information regarding other transcription factors involved in the expression of autophagy-related genes. It has been reported that the transcription factors E2F1 and NF-kB regulate Bnip3 expression^57,58^. However, activation of NF-kB has been demonstrated in the muscle of *mdx* mice^56^. Signal transducer and activator of transcription 1 (STAT1) negatively regulates autophagy by repressing the Ulk1 gene in skeletal muscle^59^ and STAT1 activity has been reported to be markedly augmented in the muscle of *mdx* mice^56^. However, since the *Becn1* gene was not altered in the skeletal muscle of STAT1 knockout mice^59^, downregulation of *Becn1* observed in *mdx* mice cannot be explained by increased STAT1 activity. It has been reported that expression of autophagy-related genes is also regulated by epigenetic mechanisms including histone modifications and non-coding RNAs. We did not examine the roles of such regulations in this study.

Another limitation is the absence of experimental results directly demonstrating that the downregulation of autophagy-related genes in the muscles of *mdx* mice is a result of phosphorylation–mediated reductions in activities of FoxOs and TFEB. In our previously study, we found that treatment of *mdx* mice with the SIRT1 activator resveratrol upregulated autophagy-related genes in the muscle^24^. In the present study, resveratrol treatment resulted in a reduction in phospho-TFEB levels in muscles from *mdx* mice. Furthermore, in a previous study, we found that resveratrol treatment increased the localization of FoxO3a in the nucleus of the hearts of *mdx* mice^47^. These findings support the notion that restoration of the activities of FoxOs and TFEB contributes to the upregulation of autophagy-related genes in the muscles of *mdx* mice, although further experiments are necessary.

In conclusion, expression of autophagy-related genes is globally downregulated in dystrophin-deficient dystrophic muscles in mice and humans. This could result from suppression of activities of FoxOs and TFEB. It is expected that correction of dysregulated transcription of autophagy-related genes may restore autophagic activity and attenuate dystrophic changes in muscles, though further studies are needed.

## Methods

### Animals

Animal experiments were approved by the Animal Care and Use Committee of Sapporo Medical University (08-100_10-118_11-046, 18-056_21-024) were performed according to the Guide for the Care and Use of Laboratory Animals (Institute of Laboratory Animal Resources, 1996) and the Animal Care and Use Committee of Sapporo Medical University. All methods were reported in accordance with the ARRIVE guidelines. Male *mdx* mice (C57BL/10ScSn-Dmdmdx/Jic) were obtained from the Central Institute for Experimental Animals (Kanagawa, Japan). Age-matched control mice (C57BL/10SnSlc) were purchased from Japan SLC, Inc. (Shizuoka, Japan). The mice were sacrificed for obtaining skeletal muscle tissue samples at 12, 22, and 71 weeks of ages. Skeletal muscle tissues were flash-frozen in liquid nitrogen and stored at − 80 °C until use.

In the present study, we analyzed tibialis anterior muscles obtained from untreated and resveratrol-treated *mdx* mice which were used in our previously study^24^. Treatment with resveratrol (0.4 g/kg food) was initiated at 9 weeks of age, and muscle tissues were sampled at 65 weeks of age^24^.

### Grip test

Forearm grip strength was evaluated by using a grip strength meter (GPM-100B, MELQUEST, Toyama, Japan). The same operator who was blinded to the genotype of the mice performed the tests. The tests were conducted at 12–13, 21–22, and 60–61 weeks of ages. We assessed grip strength power at 1-min intervals We obtained the mean value from three trials per mouse for analysis.

### Western blotting

Frozen tissues were homogenized in CellLytic MT Cell Lysis Reagent (C3228, Sigma-Aldrich, St Louis, MO, USA) with 1% Protease Inhibitor Cocktail (25,955–11, Nacalai Tesque, Kyoto, Japan) and 1% Phosphatase Inhibitor Cocktail (07,574–61, Nacalai Tesque, Kyoto, Japan) and centrifuged at 15,000 rpm for 15 min at 4 °C. The protein concentration of the supernatant was measured by using Protein Quantification Kit-Rapid (PQ01, Dojindo, Kumamoto, Japan). Supernatant fractions of equal protein concentration were boiled for 3 min at 98 °C in sample buffer (0.5 mol/l Tris HCl 5 ml, 10% SDS 8 ml, glycerol 4 ml, 2-mercaptoethanol 2 ml, BPB 0.4 mg, pH 6.8, Milli-Q 1 ml). Protein lysates loaded onto gel were transferred to PVDF membranes (#1620177, Bio-Rad, Hercules, CA, USA). The membranes were blocked with 5% bovine serum albumin (BSA) in TBS-T (50 mM Tris–HCl, 150 mM NaCl, 0.05% Tween 20) or 5% non-fat milk in TBS-T and were incubated with a primary antibody overnight at 4 °C. The membranes were washed and incubated with a secondary antibody for 1 h at room temperature. We used Immobilon Forte Western HRP substrate (WBLUF0100, Merck, Darmstadt, Germany) or Chemi-Lumi One Ultra (11644-40, Nacalai Tesque Kyoto, Japan) for detection of bands in Vilber Bio Imaging FUSION (M&S Instruments Inc, Osaka, Japan). After detection, the membranes were incubated with Re-Blot Plus Strong Solution (2504, Merck, Darmstadt, Germany) for 30 min at room temperature. Images were analyzed with ImageJ 1.53 k (National Institutes of Health). The antibodies used are listed in Supplementary Table S1.

### Analysis of gene expression by real-time quantitative PCR

Total RNA was isolated from tibialis anterior and soleus muscles using the RNeasy Fibrous Tissue Mini Kit (Qiagen, Hilden, Germany). Complementary DNA was generated from total RNA using the GoScript Reverse Transcription System (Promega, Madison, WI, USA). DNA amplification was performed by the StepOne Real-Time PCR System (Thermo Fisher Scientific, Waltham, MA, USA) using GoTaq qPCR Master Mix (A600A, Promega, Madison, WI, USA) and CXR Reference Dye (C541A, Promega, Madison, WI, USA). Each sample was run in duplicate, and the mean value was used to evaluate the mRNA levels. All data were normalized to 18 s ribosomal RNA. The primer sequences are listed in Supplementary Table S2.

### Analysis of gene expression in skeletal muscle of patients with Duchenne muscular dystrophy from the NCBI database

Differences in gene expression in healthy controls and patients with DMD were analyzed by using microarray datasets downloaded from Gene Expression Omnibus (https://www.ncbi.nlm.nih.gov/geo/). The following datasets were analyzed: GSE1004_GPL8300 (controls vs. DMD patients, source: quadriceps), GSE1007_GPL92 (controls vs. DMD patients, source: quadriceps), GSE1007_GPL93 (controls vs. DMD patients, source: quadriceps), GSE1007_GPL94 (controls vs. DMD patients, source: quadriceps) and GSE1007_GPL95 (controls vs. DMD patients, source: quadriceps). We analyzed data for which the number of absent values was less than one third of the total number of samples. We compared the mean values between controls and DMD patients. In the present study, we did not use human tissues for analyses.

### Histological analysis

Frozen tibialis anterior muscles were embedded in Tissue-Tek O.C.T Compound (Sakura Finetek USA, Torrance, CA, USA). Blocks were cross-sectioned at 10 μm by CryoStar NX50 (Thermo Fisher Scientific, Waltham, MA, USA). Sections were fixed in 40% acetone/60% methanol for 15 min at − 20 °C and then blocked with 0.3% Triton X-100, 3% BSA and 1% goat serum in PBS. Sections were incubated with primary antibodies overnight at 4 °C. The following antibodies were used for primary antibodies: rabbit polyclonal anti-TFEB (1:50 dilution; 13372-1-AP, Proteintech, Rosemont, IL, USA), rabbit monoclonal anti-FoxO1 (1:25 dilution; #2880, Cell Signaling Technology, Danvers, MA, USA), rabbit monoclonal anti-FoxO3a (1:100 dilution; #2497, Cell Signaling Technology, Danvers, MA, USA), and rat monoclonal anti-Laminin (1:50, sc-59854, Santa Cruz Biotechnology, Dallas, TX, USA). After overnight incubation, sections were probed with a secondary antibody for 90 min at room temperature. The secondary antibody used was anti-rabbit IgG antibody conjugated with Alexa488 (1:1000 dilution; A11034, Thermo Fisher Scientific, Waltham, MA, USA) and anti-rat IgG antibody conjugated with Alexa594 (1:1000 dilution; A11007, Thermo Fisher Scientific, Waltham, MA, USA). Finally, sections were stained with Hoechst33342 (1:2000 dilution; 346–07951, Dojindo, Kumamoto, Japan). Images were captured by Nikon-A1 (Nikon, Tokyo, Japan). We randomly captured 5 images from each section. Images were analyzed with ImageJ 1.53 k (National Institutes of Health).

### Statistics

All data are expressed as means ± standard deviation. Welch’s two sample t-test was used to compare average values between two groups. If the *P*-value was less than 0.05, differences were considered significant in all tests. R version 4.2.2 was used for the data analysis.

### Supplementary Information

Supplementary Information.

## Data Availability

The datasets generated and analyzed during the current study are available from the corresponding author on reasonable request. The Gene Expression Omnibus accession numbers are GSE1004 (https://www.ncbi.nlm.nih.gov/geo/query/acc.cgi?acc=GSE1004) and GSE1007 (https://www.ncbi.nlm.nih.gov/geo/query/acc.cgi?acc=GSE1007).
