# Supplementary material for: Transcriptional dysregulation of autophagy in the muscle of a mouse model of Duchenne muscular dystrophy
Source: Sci Rep. 2024 Jan 16;14:1365. doi: 10.1038/s41598-024-51746-9 (PMC10791753; doi:10.1038/s41598-024-51746-9)
Supplement: Supplementary file 1 — Supplementary Information. [file 41598_2024_51746_MOESM1_ESM.pdf]

Supplementary information

Transcriptional dysregulation of autophagy in the muscle of a mouse model of Duchenne muscular dystrophy.

Ryuta Nakashima[1], Ryusuke Hosoda[1], Yuki Tatekoshi[1], Naotoshi Iwahara[1,2], Yukika Saga[1], Atsushi Kuno*[,1]

[1]Department of Pharmacology, Sapporo Medical University School of Medicine, Sapporo, Japan.
[2]Department of Neurology, Sapporo Medical University School of Medicine, Sapporo, Japan.

*Address for correspondence:
Atsushi Kuno, MD, PhD
Department of Pharmacology
Sapporo Medical University School of Medicine
South-1, West-17, Chuo-ku, Sapporo 060-8556, Japan
Phone: +81-11-611-2111, ext.27200
Fax: +81-11-612-5861
Email: kuno@sapmed.ac.jp

Soleus (22 weeks of age)

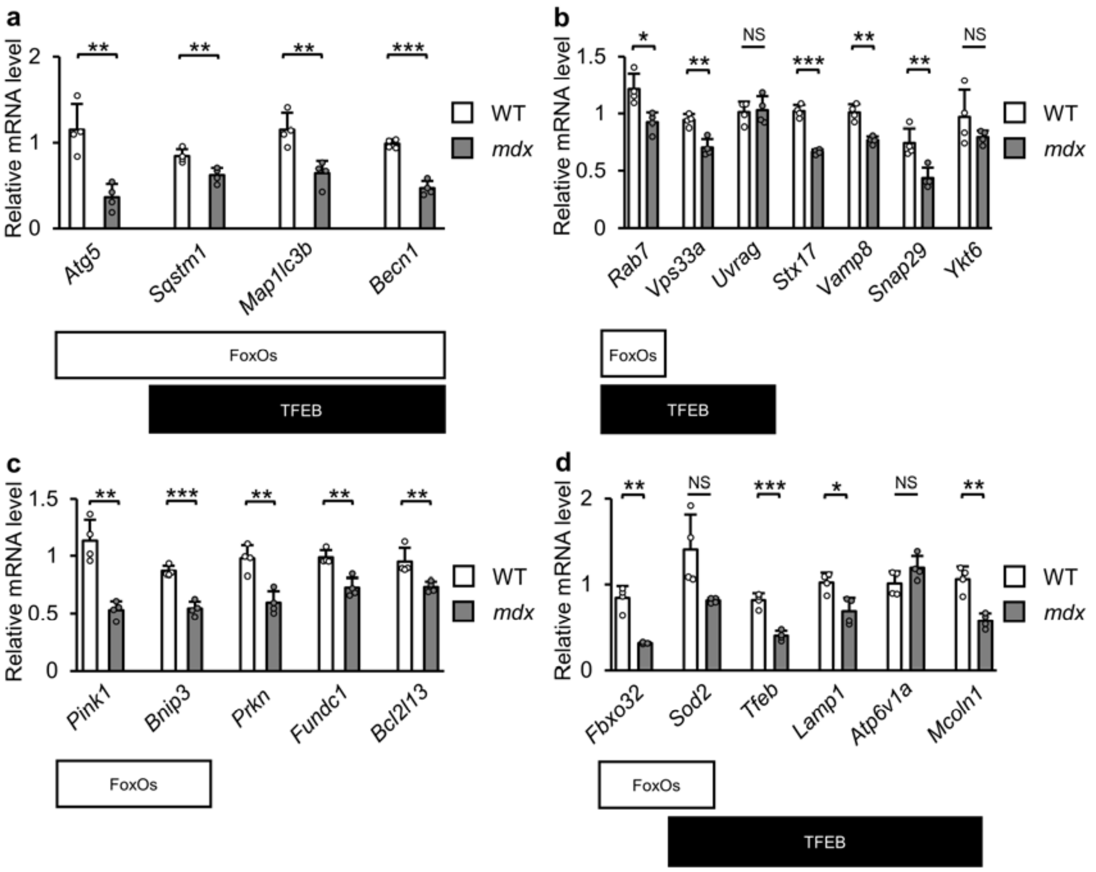

Supplementary Figure S1. Autophagy-related genes are downregulated in soleus muscles in *mdx* mice.

Expression levels of genes related to autophagosome formation **(a)**, a step of autophagosome-lysosome fusion **(b)**, mitophagy **(c)**, and the other target genes of FoxOs or TFEB **(d)** in soleus muscles of WT and *mdx* mice at 22 weeks of age (N=4). Known target genes of FoxOs and TFEB are indicated. All data are expressed as means ± standard deviation. All statistical tests were conducted by a Welch two sample t-test. *P<0.05, **P<0.01, ***P<0.001. NS, not significant.

Tibialis anterior (12 weeks of age)

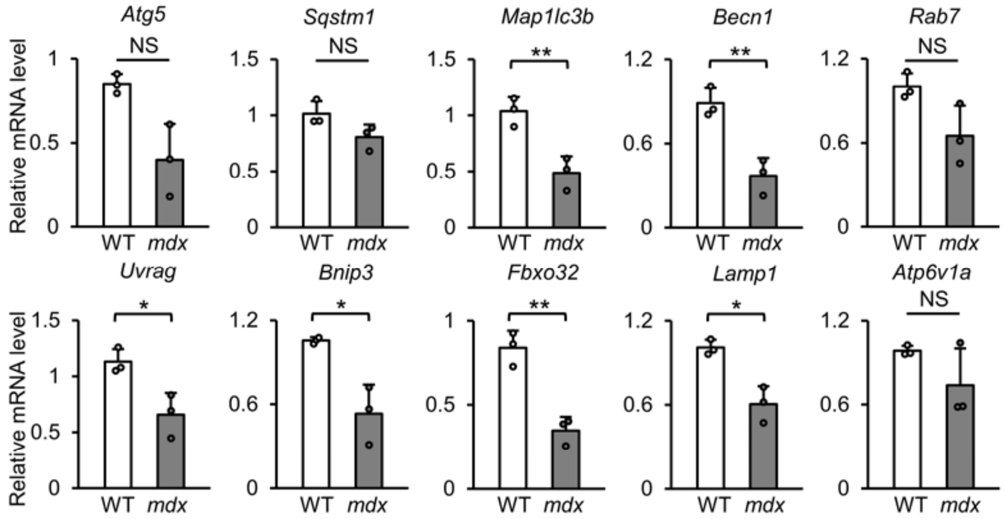

Supplementary Figure S2. Autophagy-related genes in the tibialis anterior muscle of WT and *mdx* mice at 12 weeks of age.

Expression levels of genes related to autophagy in tibialis anterior muscles of WT mice and *mdx* mice at 12 weeks of age (N=3) examined by real-time quantitative PCR. All data are expressed as means ± standard deviation. All statistical tests were conducted by Welch’s two sample t-test. *P<0.05, **P<0.01. NS, not significant.

Tibialis anterior (71 weeks of age)

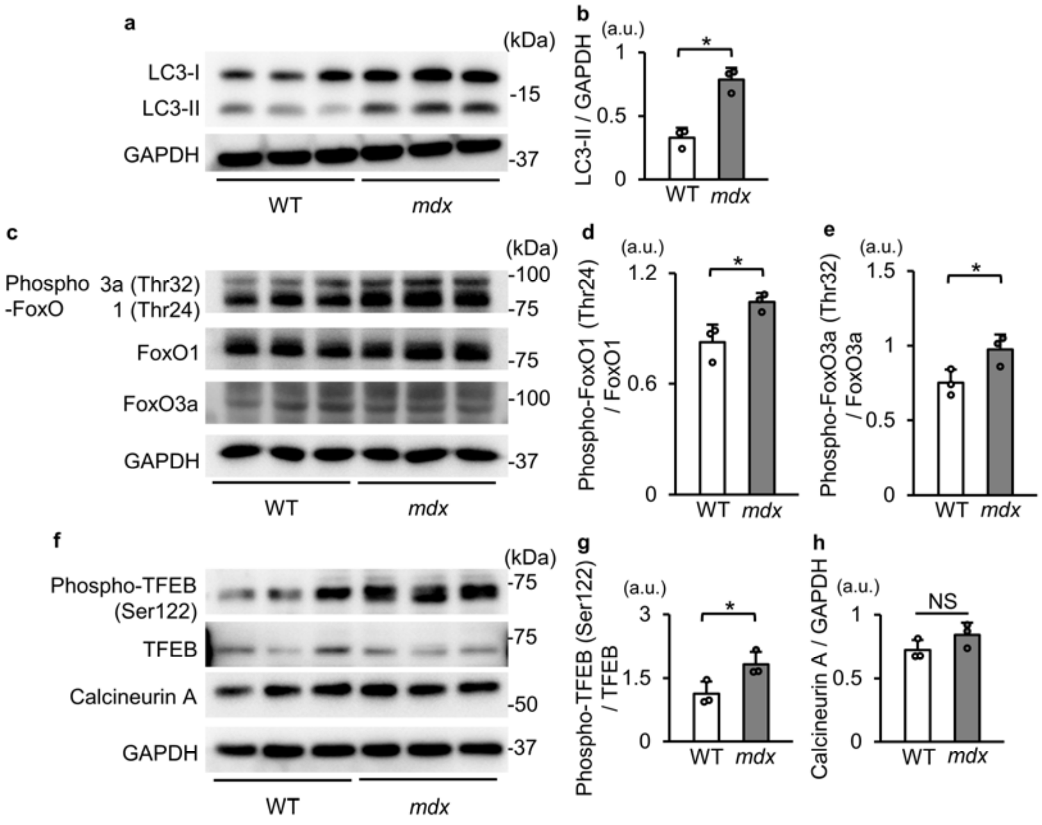

Supplementary Figure S3. Phosphorylation status of FoxO1, FoxO3a, and TFEB in WT and *mdx* mice at 71 weeks of age.

(a) Representative Western blot images for LC3 and GAPDH in tibialis anterior muscles from WT mice and *mdx* mice at 71 weeks of age. kDa: kilodalton. **(b)** Quantitative data of LC3-II levels normalized to GAPDH (N=3). **(c)** Western blot images for phospho-FoxO1 (Thr24), phospho-FoxO3a (Thr32), FoxO1, FoxO3a and GAPDH in tibialis anterior muscles from WT and *mdx* mice at 71 weeks of age. **(d)** Quantitative data of phosphorylation levels of FoxO1 normalized to FoxO1 (N=3). **(e)** Quantitative data of phosphorylation levels of FoxO3a normalized to FoxO3a (N=3). **(f)** Representative Western blot images for phospho-TFEB (Ser122), TFEB, calcineurin A, and GAPDH in WT mice and *mdx*

mice at 71 weeks of age. **(g)** Quantitative data of phosphorylation levels of TFEB normalized to TFEB (N=3). **(h)** Quantitative data of protein levels of calcineurin A normalized to GAPDH (N=3). All data are expressed as means ± standard deviation. All statistical tests were conducted by Welch's two sample t-test. *$P<0.05$. a.u.=arbitrary unit. NS, not significant.

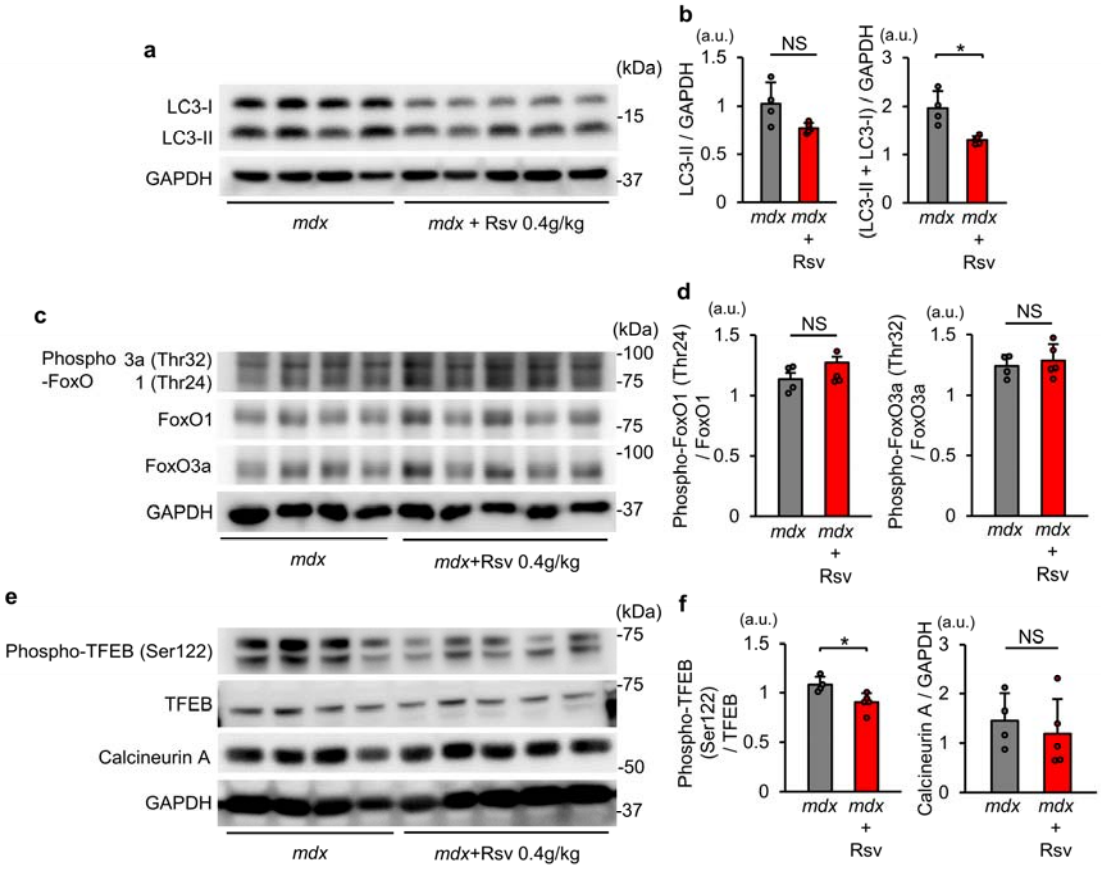

Supplementary Figure S4. Effects of resveratrol treatment on autophagic activity and phosphorylation status of FoxOs and TFEB in tibialis anterior muscles of *mdx* mice.

(a) Representative Western blot images for LC3 and GAPDH in tibialis anterior muscles from untreated and resveratrol-treated *mdx* mice. kDa: kilodalton. **(b)** Quantitative data of levels of LC3-II and LC3-I+LC3-II normalized to GAPDH (N=4 in untreated *mdx* mice, N=5 in resveratrol-treated *mdx* mice). **(c)** Western blot images for phospho-FoxO1 (Thr24), phospho-FoxO3a (Thr32), FoxO1, FoxO3a and GAPDH. **(d)** Quantitative data of phosphorylation levels of FoxO1 and FoxO3a normalized to FoxO3a (N=4-5). **(e)** Representative Western blot images for phospho-TFEB (Ser122), TFEB, calcineurin A, and GAPDH. **(f)** Quantitative data of phosphorylation levels of TFEB normalized to TFEB and protein levels of

calcineurin A normalized to GAPDH (N=4-5). All data are expressed as means ± standard deviation. All statistical tests were conducted by Welch's two sample t-test. *P<0.05, **P<0.01. NS, not significant.

Supplementary Table 1: Primary antibodies used for Western blotting.

Antibody	Source	Catalog number	Dilution
**anti-LC3A/B**	Cell Signaling Technology	#12741	1:1000
**anti-p62**	Cell Signaling Technology	#5114	1:1000
**anti-TFEB**	proteintech	13372-1-AP	1:1000
**anti-Phospho-TFEB (Ser122)**	Cell Signaling Technology	#87932	1:1000
**anti-Phospho-S6 Ribosomal Protein (Ser240/244)**	Cell Signaling Technology	#2215	1:1000
**anti-S6 Ribosomal Protein**	Cell Signaling Technology	#2217	1:1000
**anti-GAPDH**	Sigma-Aldrich	G8795	1:40000
**anti-Phospho-FoxO1 (Thr24)/FoxO3a (Thr32)**	Cell Signaling Technology	#9464	1:1000
**anti-FoxO1**	Cell Signaling Technology	#2880	1:1000
**anti-FoxO3a**	Cell Signaling Technology	#2497	1:1000
**anti-Pan-Calcineurin A**	Cell Signaling Technology	#2614	1:1000
**anti-Phospho-Akt (Ser473)**	Cell Signaling Technology	#9271	1:1000
**anti-Akt**	Cell Signaling Technology	#4691	1:1000

Supplementary Table 2: Primer sequences used for real-time quantitative PCR.

Gene	Forward	Reverse
18s	5'-CGGACAGGATTGACAGATTG-3'	5'-CAAATCGCTCCACCAACTAA-3'
Becn1	5'-CTGACAGACAAATCTAAGGAG-3'	5'-AATAGGAGCCGCCACTGCCTC-3'
Atg5	5'-TTGGAACATCACAGTACATTT-3'	5'-AACGAAATCCATTTTCTTCTG-3'
Map1lc3b	5'-CGTCCTGGACAAGACCAAGT-3'	5'-ATTGCTGTCCCGAATGTCTC-3'
Sqstm1	5'-CCTTGCCCTACAGCTGAGTC-3'	5'-CACACTCTCCCCCACATTCT-3'
Tfeb	5'-AACAGTGCTCCCAACAGTCC-3'	5'-GGCGCATAATGTTGTCAATG-3'
Lamp1	5'-ACATCAGCCCAAATGACACA-3'	5'-GGCTAGAGCTGGCATTCATC-3'
Pink1	5'-TGAGGAGCAGACTCCCAGTT-3'	5'-AGTCCCACTCCACAAGGATG-3'
Prkn	5'-TGGAAAGCTCCGAGTTCAGT-3'	5'-CCTTGTCTGAGGTTGGGTGT-3'
Bnip3	5'-TCCACTAGCACCTTCTGATGA-3'	5'-GAACACCGCATTTACAGAACAA-3'
Fundc1	5'-CCCCCTCCCCAAGACTATGAA-3'	5'-CCACCCATTACAATCTGAGTAGC-3'
Bcl2l13	5'-CTCAGCCAGCAGTGACATA-3'	5'-GGCACCAAAAGCTTATTCCA-3'
Rab7	5'-TGAACCCATCAAACTGGACA-3'	5'-GAGGAGGGACGCATATTGAA-3'
Vps33a	5'-GAAGGGAGATCCTCCAGACC-3'	5'-GCAGTGTTTTCCGGATTGTT-3'
Stx17	5'-GTGAAAGCCTGGAGCAAGAC-3'	5'-TGTGTAAACACCTGGGAGCA-3'
Snap29	5'-AACCAGTAGAGCCTCCACCT-3'	5'-CTTCTGAGGTTTGGGTGGCT-3'
Vamp8	5'-AGAATGTGGAGCGGATCTTG-3'	5'-AGTGTTCAGACGTGGCTTCC-3'
Ykt6	5'-AAAGGCAGCAGAGCTTCTGT-3'	5'-GCTGTCAGCAATGACCACAC-3'
Atp6v1a	5'-TCGGAAACCCTGAGAGAGA-3'	5'-CTTATCCAAGCCCCAGAACA-3'
Uvrag	5'-TGGTGCTTCGAAATGAACTG-3'	5'-CACTCAGGGAGTCCTTCTGC-3'
Fbxo32	5'-CTTTCAACAGACTGGACTTCTCG-3'	5'-CAGCTCCAACAGCCTTACTACGT-3'
Sod2	5'-GACCCATTGCAAGGAACAA-3'	5'-GTAGTAAGCGTGCTCCCACAC-3'
Mcoln1	5'-GTCGGTGTCATTCGCTACCTGA-3'	5'-GAACGATCCAGCCACAGAAGCA-3'
